# The Etiology of Gastric Ulcer

**Published:** 1888-02

**Authors:** 


The Etiology of Gastric Ulcer.—Impressed by the fact that women employed as cooks so frequently suffer from gastric ulcer, Decker (*Fortschritte der Med.*, B. v., 415,) instituted some experiments on the action of hot foods in producing the disease. Two dogs were repeatedly fed through the stomach tube with semi-solid food heated to 120° F. The first received nourishment in this way four times, and the other eight times. At the autopsy of the first dog, the mucous membrane of the stomach appeared normal in all parts, except at the lesser curvature, where there was situated a hyperæmic spot, about four-tenths of an inch in diameter, caused by a hemorrhagic extravasation between the mucous and muscular layers.

In the second dog, there was found a dark red area on the posterior gastric wall, about the size of a quarter of a dollar. The mucous membrane over it was shriveled, and resembled felt, and had been somewhat separated from the muscular layer by the occurrence of hemorrhage between them. In the pyloric region were two typical gastric ulcers, extending to the serous layer. The author considers the hemorrhagic infiltration, the shrinking and elevation of the mucous membrane, and its final destruction, to be the three phases in the formation of gastric ulcer.

